# EUS-guided gastroenterostomy to treat gastric outlet obstruction in a patient with gastric lymphoma followed by pyloric recanalization using a rendezvous technique

**DOI:** 10.1016/j.vgie.2021.01.004

**Published:** 2021-02-27

**Authors:** Michael Lajin

**Affiliations:** Sharp Grossmont Hospital, La Mesa, California

**Keywords:** LAMS, lumen-apposing metal stent, TB, tuberculosis

## Abstract

Video 1 EUS-guided gastroenterostomy to treat gastric outlet obstruction followed by pyloric recanalization using a rendezvous technique

EUS-guided gastroenterostomy to treat gastric outlet obstruction followed by pyloric recanalization using a rendezvous technique

## Introduction

The rendezvous technique was described to salvage a dislodged stent during EUS-guided gastrojejunostomy.^1^ We describe using this technique to perform endoscopic gastroenterostomy because of the unsafety of traditional methods^2^ in this case.

## Case

**Figure 1:**
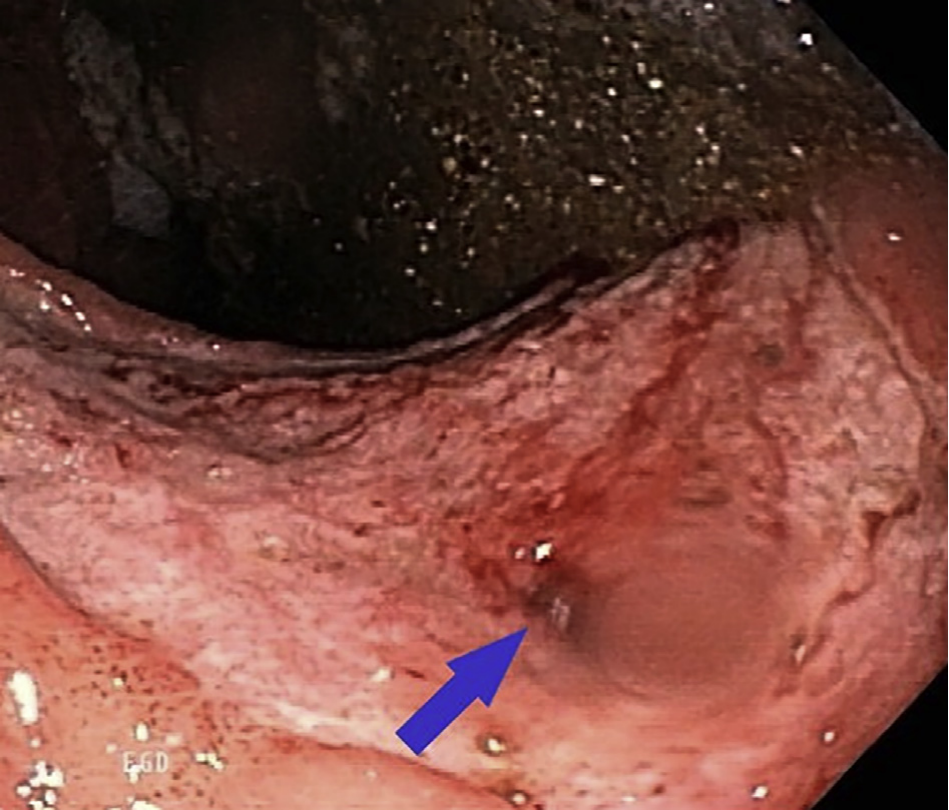
Large antral ulcer involving the pylorus (*blue arrow*), resulting in outlet obstruction.

A 66-year-old man was diagnosed with gastric lymphoma and received chemotherapy. He subsequently developed pulmonary tuberculosis (TB) and started TB medications. He presented with vomiting and 80-pound weight loss. Endoscopy revealed a pyloric ulcer resulting in outlet obstruction (Fig. 1) and proximal gastric ulcers. Attempted placement of a jejunal extension to a percutaneous gastrostomy tube by interventional radiology was unsuccessful due to severe pyloric narrowing. He was deemed a poor surgical candidate and remained on total parenteral nutrition and in respiratory isolation (Video 1, available online at www.VideoGIE.org).

## Procedure

**Figure 2:**
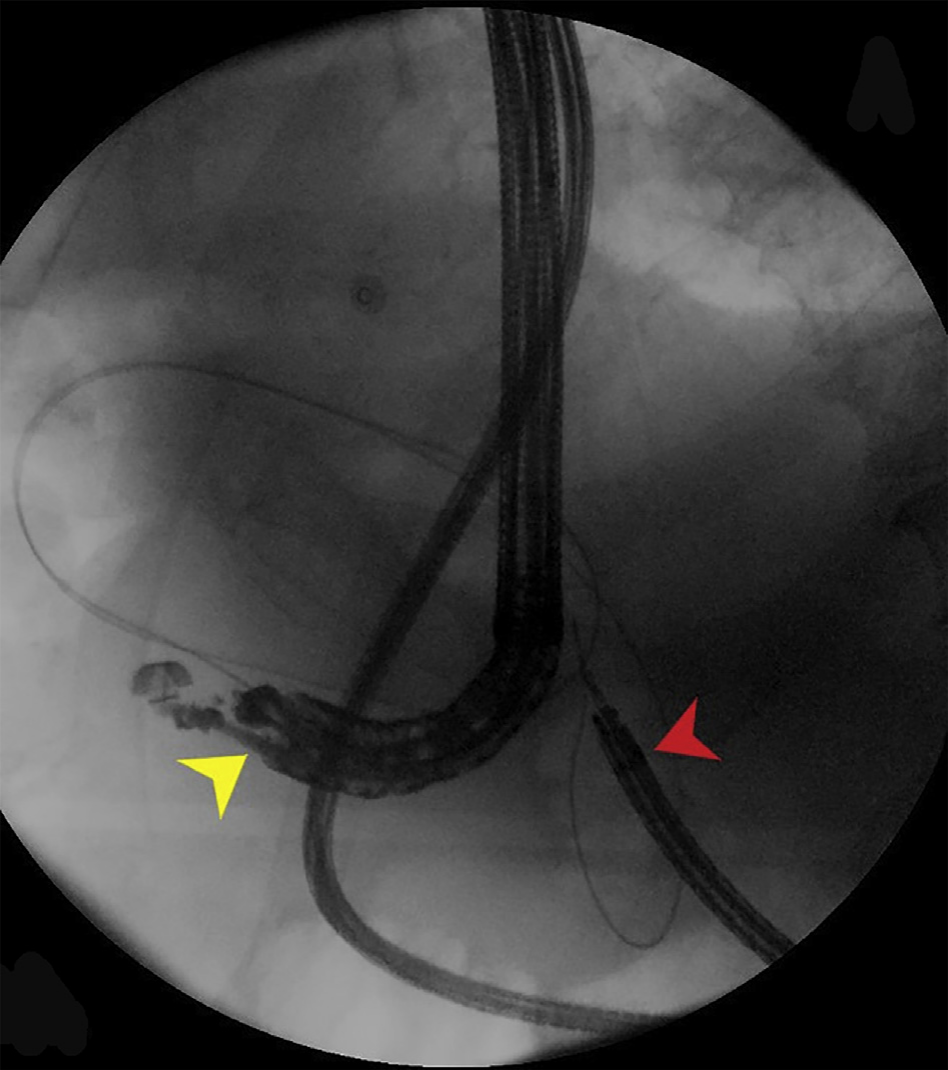
Under guidance from EUS positioned in the stomach (*yellow arrowhead*), a wire was advanced to the jejunum and was grasped by the ultraslim endoscope (*red arrowhead*) positioned in the proximal jejunum.

**Figure 3:**
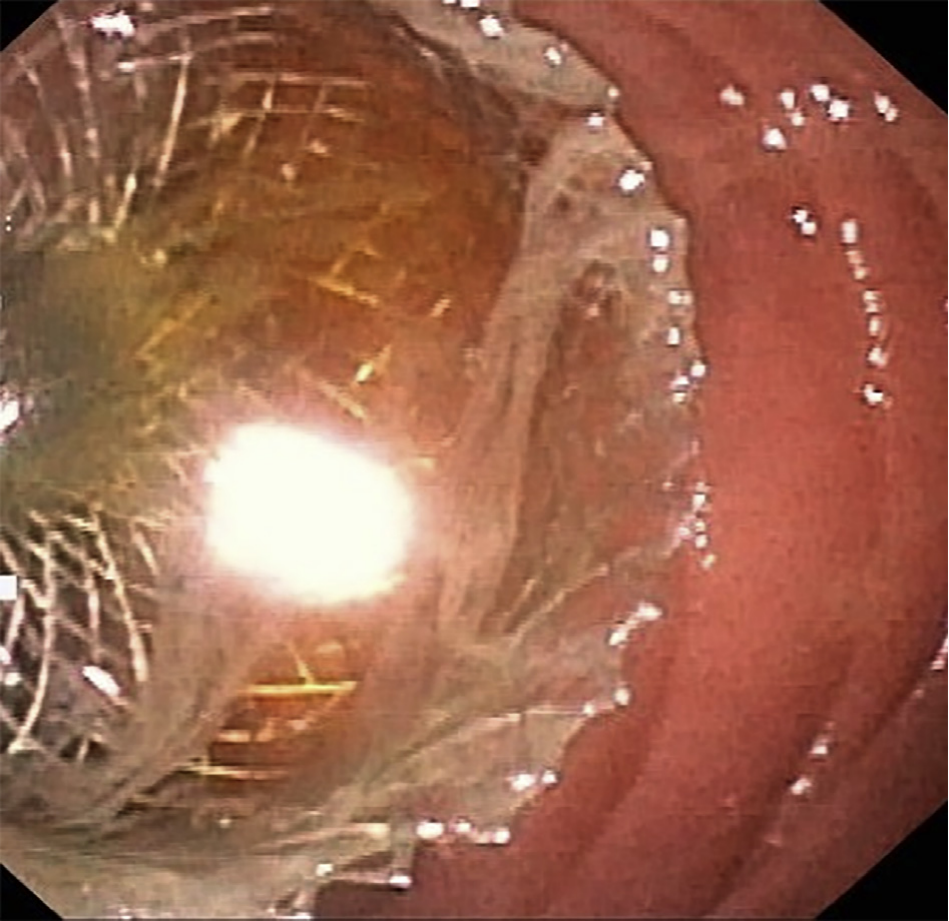
Deployment of the distal flange of the lumen-apposing metal stent in the jejunal lumen was verified by the ultraslim endoscope positioned in the jejunum.

**Figure 4:**
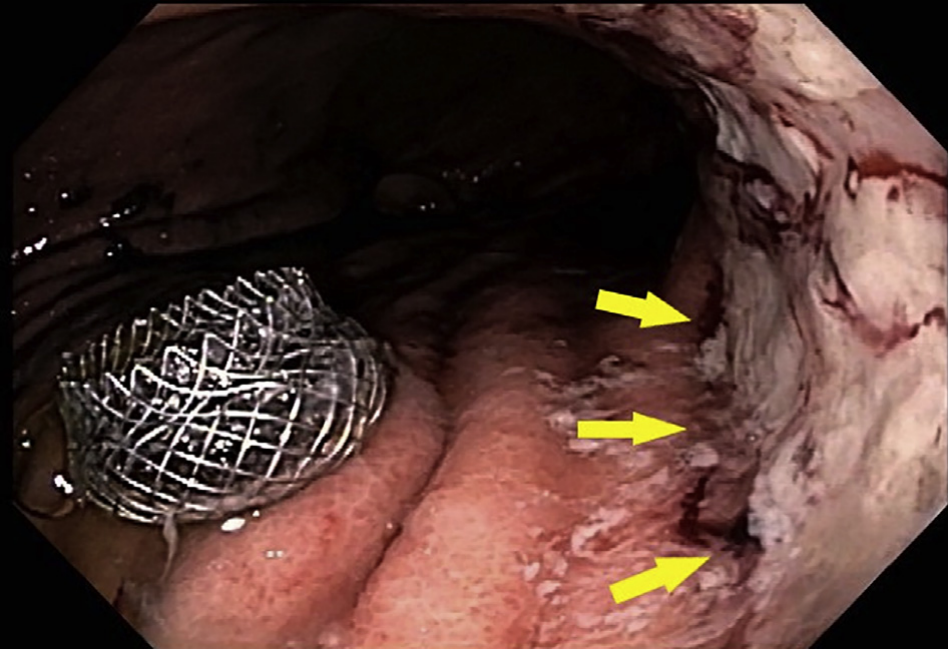
Endoscopic view of lumen-apposing metal stent after deployment and the nearby proximal gastric ulcer (*yellow arrows*).

A regular endoscope could not traverse the pylorus. A transpyloric stent and/or dilation carries a risk of bleeding or perforation. The pylorus was traversed using an ultraslim endoscope that was used to distend the target jejunal loop with contrast. A linear echoendoscope was advanced on the side of the endoscope to the stomach. After identifying the target jejunal loop on ultrasound, the site of the planned gastroenterostomy seemed in close proximity to the proximal gastric ulcer. Because our echoendoscope was not forward-viewing, we decided against direct deployment of lumen-apposing metal stents (LAMSs) to avoid going through the ulcer. A 19-gauge needle was used to puncture the targeted jejunum, and a long wire was advanced (Fig. 2). The wire was pulled by the endoscope to the mouth. Care was taken to avoid wire entanglement. The echoendoscope was removed. A therapeutic endoscope was advanced over the gastric end of the wire. The wire entry was confirmed to be clear from the ulcer. The track was dilated with a 6-mm hurricane balloon. A 2-cm LAMS was deployed, using the ultraslim endoscope to confirm the deployment of the distal flange inside the jejunal lumen (Figs. 3 and 4). The stent was dilated to 2 cm.

**Figure 5:**
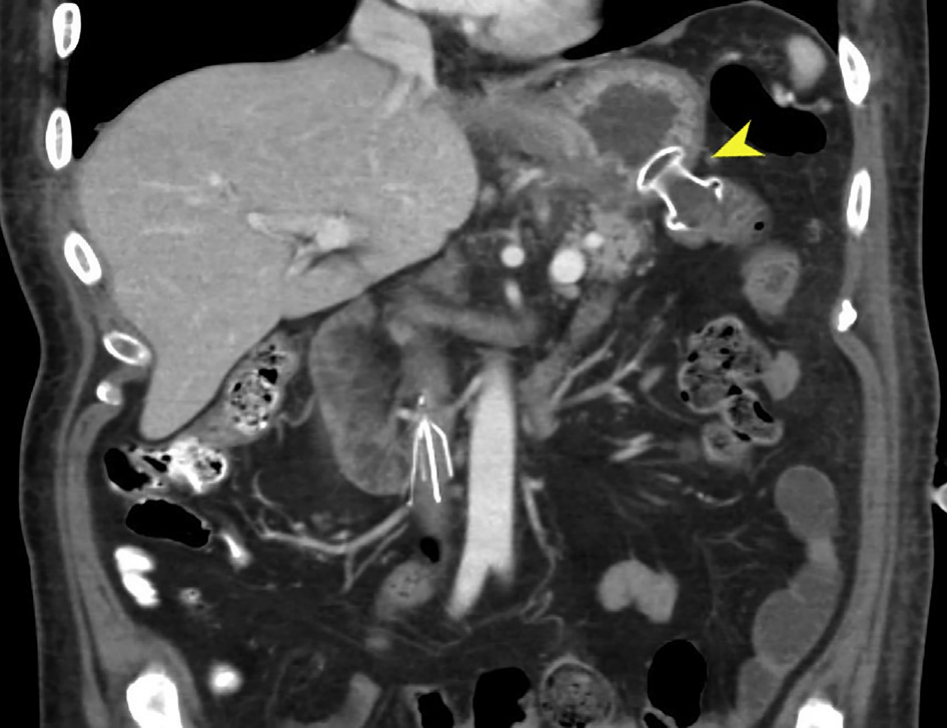
Postoperative CT image of the gastroenterostomy (*yellow arrowhead*).

**Figure 6:**
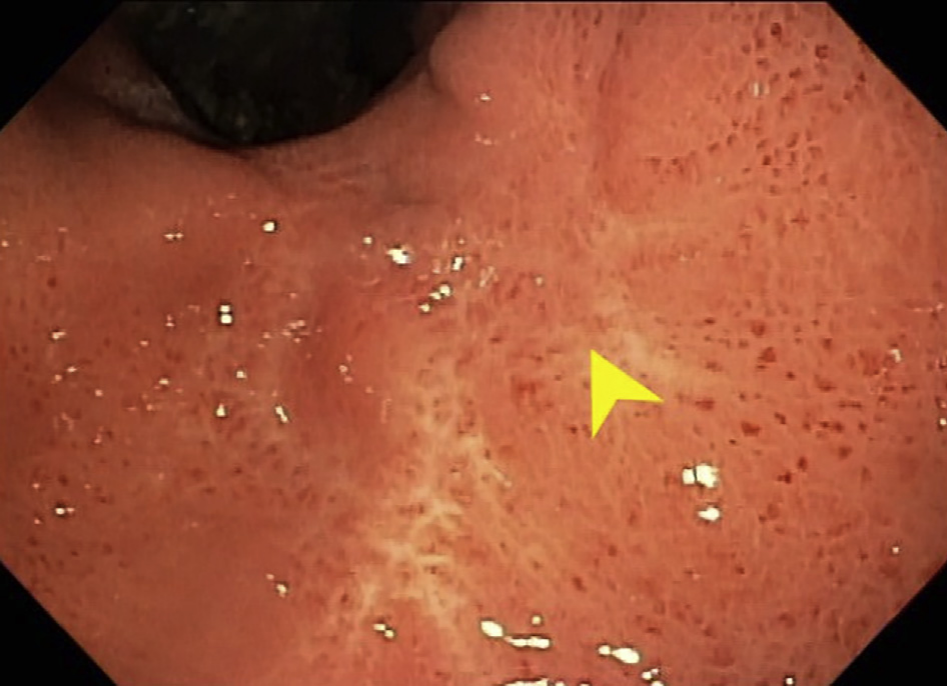
Complete closure of the pylorus (*yellow arrowhead*) after healing from the ulcer.

Postoperative CT revealed gastric decompression (Fig. 5). Oral diet and TB medication were resumed, and the patient was eventually discharged. Four months later, he regained the lost weight and was clear from TB and lymphoma. Endoscopy confirmed ulcer healing resulting in complete closure of the pylorus (Fig. 6).

Long-term options were discussed. The patient declined surgery. Removal of LAMSs will lead to fistula closure. Keeping LAMSs long term, however, will result in tissue growth and degradation of the silicone. Our options were either periodic LAMS exchange or pyloric recanalization.^3^ He elected to proceed with pyloric recanalization.

**Figure 7:**
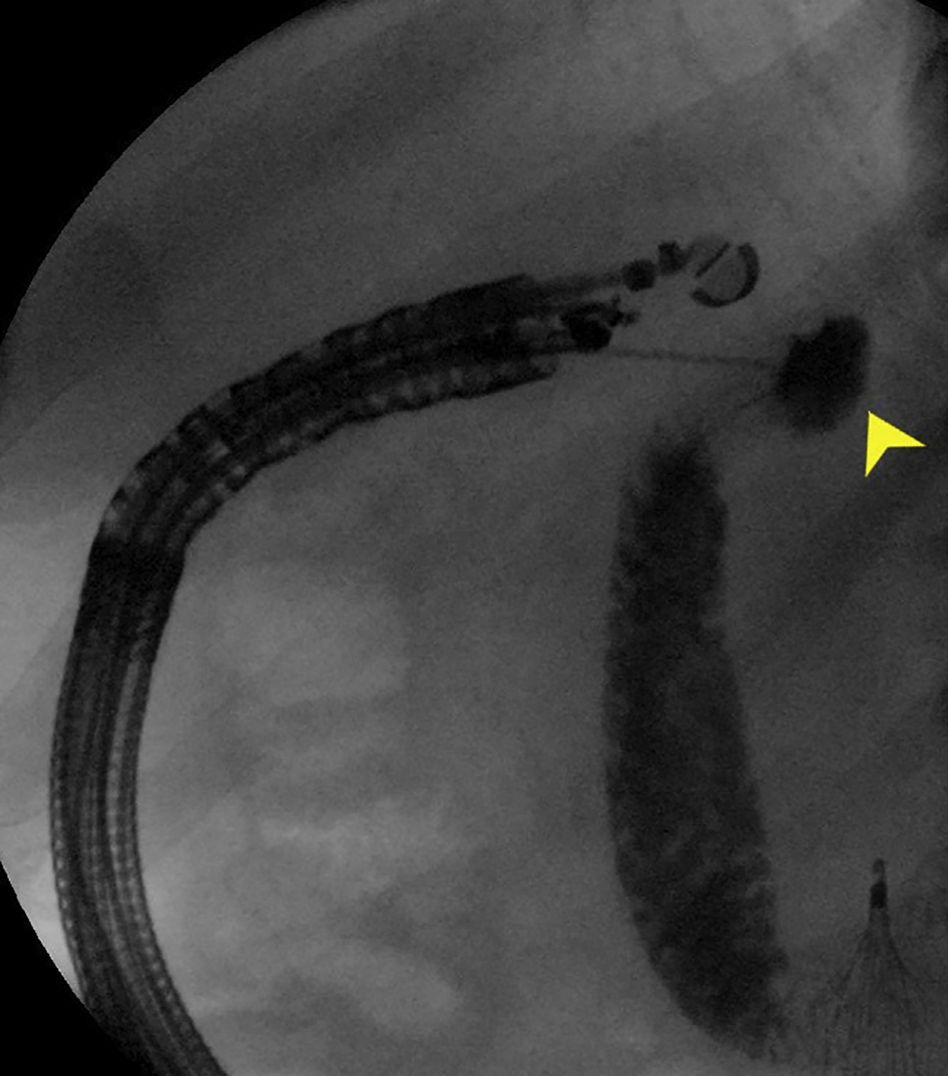
Contrast injected through the FNA needle without adequate distension of the duodenal bulb (*yellow arrowhead*).

**Figure 8:**
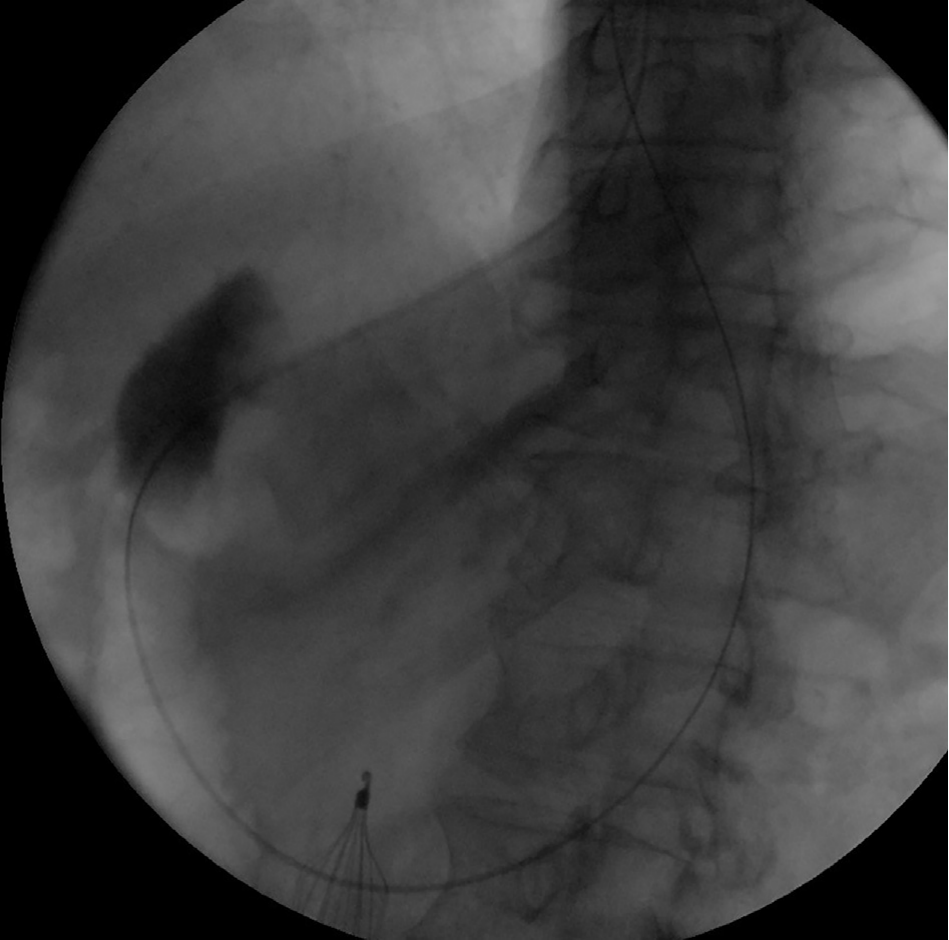
Fluoroscopic image of the wire after controlling the 2 ends of the wire at the mouth.

**Figure 9:**
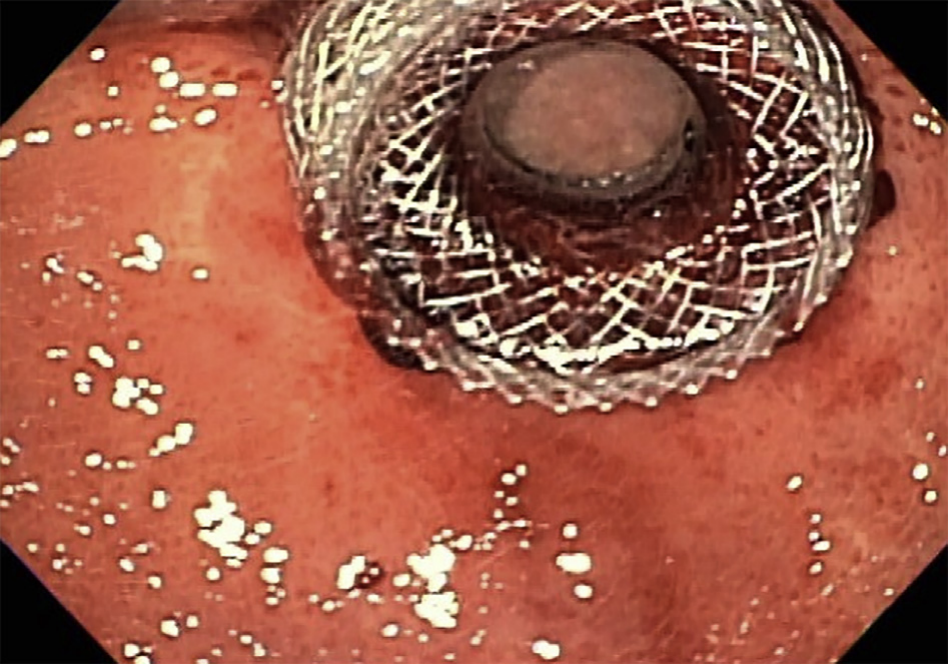
Endoscopic view of lumen-apposing metal stent after gastroduodenostomy.

The echoendoscope was advanced to the antrum. The duodenal bulb was punctured by using a 19-gauge needle. In spite of using a pump to inject contrast through the needle, the bulb did not distend adequately, prohibiting direct LAMS deployment (Fig. 7). A long wire was passed to the duodenum. The echoendoscope was removed while keeping the wire in place. A pediatric colonoscope was advanced through the gastroenterostomy to the proximal duodenum. The wire was pulled by the colonoscope to the mouth (Fig. 8). Care was taken to avoid wire entanglement. A therapeutic endoscope was advanced over the gastric end of the wire to the pylorus. A hurricane balloon catheter was advanced through the scar. This was facilitated by gently pulling the duodenal end of the wire at the mouth. The track was dilated to 8 mm. A 2-cm LAMS was deployed across the pylorus without using electrocautery and dilated to 15 mm (Fig. 9).

## Outcome

There were no adverse events. Eight weeks later, the pyloric stent was removed. Two months after stent removal, the pylorus was patent and was dilated to 15 mm. Our plan is a gradual pyloric dilation and eventual removal of the gastroenterostomy stent once long-term pyloric patency is confirmed.

## Conclusions

The rendezvous technique can be used to perform an endoscopic gastroenterostomy when a forward view is needed to ensure a safe path or when the target loop is not adequately distended to allow a direct approach.

## Disclosure

*The author disclosed no financial relationships.*

## Supplementary data

Video 1 EUS-guided gastroenterostomy to treat gastric outlet obstruction followed by pyloric recanalization using a rendezvous technique
